# Portable Sensors Add Reliable Kinematic Measures to the Assessment of Upper Extremity Function

**DOI:** 10.3390/s19051241

**Published:** 2019-03-12

**Authors:** Fredrik Öhberg, Tomas Bäcklund, Nina Sundström, Helena Grip

**Affiliations:** Department of Radiation Sciences, Biomedical Engineering, Umeå University, 901 87 Umeå, Sweden; Fredrik.Ohberg@umu.se (F.Ö.); Nina.Sundstrom@vll.se (N.S.); Helena.Grip@vll.se (H.G.)

**Keywords:** inter-rater reliability, inertial sensor, kinematics, upper limb, arm function

## Abstract

Ordinal scales with low resolution are used to assess arm function in clinic. These scales may be improved by adding objective kinematic measures. The aim was to analyze within-subject, inter-rater and overall reliability (i.e., including within-subject and inter-rater reliability) and check the system’s validity of kinematic measures from inertial sensors for two such protocols on one person. Twenty healthy volunteers repeatedly performed two tasks, finger-to-nose and drinking, during two test sessions with two different raters. Five inertial sensors, on the forearms, upper arms and xiphoid process were used. Comparisons against an optical camera system evaluated the measurement validity. Cycle time, range of motion (ROM) in shoulder and elbow were calculated. Bland–Altman plots and linear mixed models including the generalizability (G) coefficient evaluated the reliability of the measures. Within-subject reliability was good to excellent in both tests (G = 0.80–0.97) and may serve as a baseline when assessing upper extremities in future patient groups. Overall reliability was acceptable to excellent (G = 0.77–0.94) for all parameters except elbow axial rotation in finger-to-nose task and both elbow axial rotation and flexion/extension in drinking task, mainly due to poor inter-rater reliability in these parameters. The low to good reliability for elbow ROM probably relates to high within-subject variability. The sensors provided good to excellent measures of cycle time and shoulder ROM in non-disabled individuals and thus have the potential to improve today’s assessment of arm function.

## 1. Introduction

Gait analysis is well-established in clinical assessment of movement disabilities [1] but motion analysis in the evaluation of upper extremity function is less common but equally important in several neurological and musculoskeletal disorders, e.g., stroke [2,3,4], Parkinson’s disease [5] and shoulder instability [6]. Information about the upper extremities’ function can help to determine the extent of a problem and guide the clinician to design and evaluate an intervention. To fit the needs of today’s health care, assessment procedures need to be valid, reliable and rater independent, time-effective and easy to perform.

Neurological dysfunctions affect the activities of daily living (ADL), hence standardized tests, e.g., the modified Ashworth scale of muscle spasticity [7], are important in the clinic. Drawbacks with such scales are that they do not give explicit information about the movement quality and may be insensitive to small and more specific changes [8]. One common ADL task is the drinking task, which is a natural and purposeful task to assess the function of the arm when grasping and lifting a glass of water, taking a sip and putting the glass down again. Recent studies point out that kinematic measures of the drinking task give additional and more sensitive information about movement function [9,10,11]. It is also important to measure movement quality, e.g., a study showed that persons with stroke had excessive arm-trunk movements during a standing coordination test and that they could not perform fast movements with the same movement quality as a healthy control group [12]. To quantify such changes in movement capacity, kinematic measures might be valuable [13].

Arm function in persons with neurological dysfunction is also routinely evaluated using ordinal scales such as the Fugl-Meyer assessment [14,15]. The finger-to-nose task is part of the Fugl-Meyer assessment, and it aims to evaluate coordination and speed by measuring and comparing, for instance, total performance time between the affected and non-affected arm [16]. To individualize the rehabilitation program and track changes in a movement deficit over time, it is important to have other outcome measures apart from performance time that describes the quality of the movement. The challenge is to develop movement analysis methods that can be used in the clinical environment. Simple equipment such as goniometers, measuring the static angle in one plane, can be used to measure the range of motion in flexion/extension/rotation [15,17], but by introducing inertial motion sensors these and several other parameters can be quantified objectively and over time. Inertial motion sensors have been used to e.g., classify arm movement features during ADL activities [18] or to construct a portable rehabilitation system for patients/individuals with brain injury [19]. The sensors are easy to use, but to be applicable for evaluation of arm function in clinical practice, the within-subject and inter-rater reliability of the specific protocols also needs to be confirmed. One way to study reliability is to use the theory of generalizability. Here, multiple sources of measurement errors are used to characterize a measurement, as compared to classical test theory where instead undifferentiated random errors are modeled [20]. This gives a more detailed description of how different types of errors impact the overall reliability. It also enables calculation of the number of repetitions needed to optimize the reliability of a certain task. The question is whether kinematic outcome measures, based on inertial sensors, can be incorporated in a protocol that assesses the arm function in a reliable way.

The aim of this study was to analyze within-subject, inter-rater and overall reliability (i.e., including within-subject and inter-rater reliability) for joint rotations and temporal outcome measures of the finger-to-nose and drinking task using a portable inertial motion sensor system. The second aim was to do a preliminary validation of the system on one person against a gold standard optical 3D motion capture system.

## 2. Materials and Methods

### 2.1. Participants

Twenty healthy volunteers (39.8 ± 11.6 years (mean ± standard deviation (SD)), seven women), height 1.74 ± 0.09 m and Body Mass Index 24.85 ± 2.4 kg/m^2^ participated in this study. The participants had normal function in their back and upper extremities, i.e., no reported neurological conditions or reduced ability to perform daily routines and were recruited among personnel at University Hospital of Umeå, Sweden. The study was conducted in accordance with the Declaration of Helsinki, and the study was approved by the Regional Ethical Review Board in Umeå (Dnr 09–120 M). Written informed consent was obtained from each participant.

### 2.2. Measurements

The portable movement analysis system included five tri-axial inertial motion sensors (MoLab^TM^ POSE, AnyMo AB, Umeå, Sweden) connected by flexible cables to a data acquisition unit, which wirelessly transmitted data to a laptop where it was registered with the software MoLab^TM^ Measure. Each sensor was 60 × 45 × 10 mm, included a 3D gyroscope, accelerometer, and magnetometer and weighted 14 g. Sampling frequency was 128 Hz (16-bit resolution, gyroscopes with full-scale range ±1000°/s, accelerometers ±8 g and magnetometers ±4800 µT). Details about the signal processing of the inertial sensor data are described in an earlier study [21]. Measurement precision and accuracy of this system were previously validated against a gold standard optical system during standardized arm tasks (cone picking, throwing and two different coordination tasks) in ten healthy volunteers and one participant with sequel after stroke. This study showed that the system’s systematic error ranges between −1.2° and 2.0° [22]. A similar setup during leg movements in five healthy participants showed precision and accuracy of 2–3° [21]. El-Gohran and McNames validated a similar sensor against a high-precision robot arm which attained an RMS angle error of about 3° for all measured angles during slow, normal and fast movements [23]. The measurement error was evaluated by comparisons of the portable system against a gold standard optical system (Qualisys Track Manager, Qualisys AB, Sweden) on one subject.

### 2.3. Study Design and Test Protocols

Each participant performed two tasks: finger-to-nose and drinking. In the finger-to-nose task, participants were asked to touch the nose with the index finger and then return the hand to the initial position on the table in front of him/her. Each movement was performed 10 times with eyes open at self-selected speed. In the drinking task, participants were asked to lift a glass filled with 2 dl of water, take a small sip, put the glass down and then return to the initial position at self-selected speed. The glass was placed along the midline 30 cm in front of the participant. To exclude dependency on the distance chosen, the test was also repeated placing the glass at 7 and 50 cm. Ten repetitions were performed at each distance at self-selected speed. Verbal instructions were recorded and played back to the participant before the tests started. Two different raters, with different background and experience in collecting movement data (one with long and one without any previous experience in using motion analysis systems) performed all measurements on each of the 20 participants. The rater who would start taking measurements was randomly chosen for each participant. Both tests were performed with each arm, in a randomized order, and with no rest in between.

**Figure 1:**
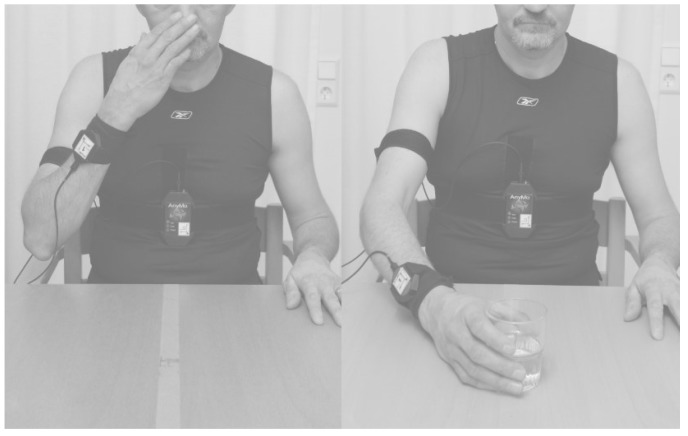
The picture shows sensor placement on one arm and on the xiphoid process.

The participant sat on a chair with hips and knees flexed 90° and feet firmly placed on the floor in front of an adjustable table. The upper arm was initially oriented vertically along the body with the elbow flexed 90°, the palm placed on the table and the forearm parallel with the body’s sagittal plane. Two sensors were placed on each forearm, dorsally and with their center 5 cm proximally from the ulnar styloid processes, two on the upper arms, dorsally and with their center 10 cm proximally from the olecranon, and one on the upper body on the xiphoid process. All sensors were attached with custom made Velcro straps, illustrated in Figure 1. All sensors were removed from the participant before the subsequent rater started the second measurement session approximately five minutes after the first.

A few participants did less than ten repetitions by mistake, and data collected from one participant’s non-dominant arm during one test session were excluded because one sensor came loose. In total, 776 and 2321 observations in the finger-to-nose and drinking task were analyzed. 

### 2.4. Data Processing

**Figure 2:**
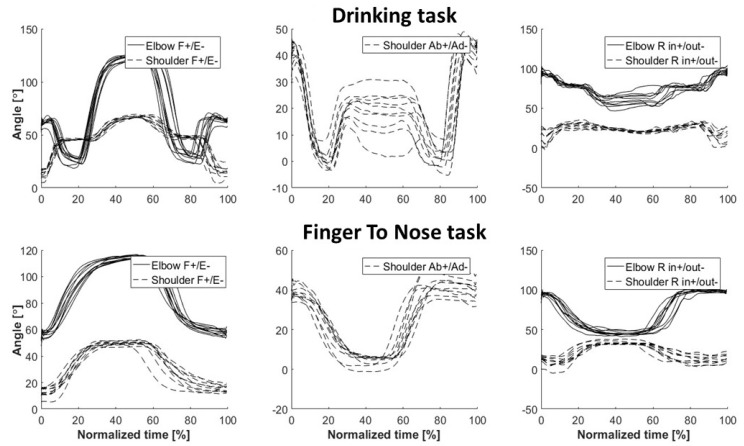
Examples of a subject’s movement pattern during the finger-to-nose and drinking tasks for one rater. The patterns are illustrated using a normalized time window that was defined by the beginning and end of each repetition, giving 10 movement curves for each test. The illustration from the drinking task is collected from the test where the glass was placed 30 cm in front of the subject. The abbreviations are F (flexion; positive angles), E (extension; negative angles), Ab (abduction; positive angles), Ad (adduction; negative angles), R in (pronation(elbow); inward humeral rotation (shoulder); positive angles) and R out (supination (elbow); outward humeral rotation (shoulder); negative angles).

The software MoLab^TM^ Analysis was used for data processing and analysis. Start and stop events for each repetition were manually determined from observation of joint angular curves and used for time normalization (Figure 2). Each body segment’s orientation in a global space was defined by calculating the orientation of the motion sensor relative to a global coordinate system, using the gravity vector from the accelerometer, the compass direction from the magnetometer and the cross product between those two vectors. Quaternions were calculated for each time frame and then transposed into elbow and shoulder joint angles. Each joint angle was defined as the rotation of the distal segment relative to the proximal segment using the Cardan X-Y-Z convention; +X represented an extension, +Y adduction and +Z inward axial rotation, adapted from the ISB recommendations [24]. To determine the system’s validity, the portable system’s measurement error against the optical system was evaluated for one subject. Quaternions were calculated for each rigid marker cluster. The helical angle during movement in relation to the initial resting position was then compared between the systems for each body segment and task [25].

ROM was calculated for the shoulder and elbow joints for each repetition in the tests. As the shoulder is a ball-and-socket joint, ROM was given for *flexion-extension* (FE), *abduction/adduction* (AbAd) and *inward/outward axial rotation* (R). The elbow hinge joint allows flexion-extension and axial rotation (positive angle pronation, negative angle supination) of the forearm relative to the upper arm, hence FE and R were investigated. A temporal measure describing the time in seconds for each repetition, cycle time, was also calculated.

### 2.5. Statistics

R-studio (version 1.0.143) and R (version 3.3.3) were used for all statistical analyses and three different approaches were applied: linear regression, Bland–Altman plots and linear mixed models (LMM) including calculation of the generalizability (G) coefficient [26]. Linear regressions and Bland–Altman plots, analyzed for each outcome measure and test, were used to illustrate the agreement between measures made by the two different raters (calculated using package ‘BlandAltmanLeh’, version 0.3.1). Bland–Altman plots were also used to compare the inertial and optical sensor systems to verify the system´s validity.

For the LMM, R-package ‘lme4′ version 1.1.12 was used. The response measure was set to ROM or cycle time. The random effect was a mix of subject, rater and repetition identifiers including all two-way interactions. 

The dataset was stratified using the factor arm dominance since clinical investigations are likely to be performed mostly on one specific arm (dominant/non-dominant, injured/non-injured). Thus, estimates of the measurement variance (divided into six random factors) were calculated, i.e., between subjects (σ^2^_sub_); between repetitions (σ^2^_rep_); between raters (σ^2^_rat_); subject/rater interactions (σ^2^_subj_rat_); subject/repetition interactions (σ^2^_subj_rep_) and rater/repetition interactions (σ^2^_rat_rep_) as well as residuals (σ^2^_residual_).

For calculating the G-coefficients, the facets considered were rater and repetition in a crossed design with an absolute agreement. Equations (1) to (3) were applied for estimating reliability (i.e., within-subject), inter-rater reliability (between raters) and overall test reliability (total test situation including two raters, n_rat_ = 2, and 10 repetitions, n_rep_ = 10) respectively [26]. The term within-subject reliability, instead of within-subject variability, was used to avoid confusion with variance. For studying the effect of repeated measurements, Equation (3) with n_rat_ = 1 was used. As thresholds, G > 0.90 indicate excellent reliability, 0.80–0.89 good reliability and 0.70–0.79, acceptable reliability [27].

(1) $$G_{rep}=\frac{\sigma_{subj}^{2}+\sigma_{rat}^{2}+\sigma_{subj\_rat}^{2}}{\sigma_{subj}^{2}+\sigma_{rat}^{2}+\sigma_{rep}^{2}+\sigma_{subj\_rat}^{2}+\sigma_{subj\_rep}^{2}+\sigma_{rat\_rep}^{2}+\sigma_{residual}^{2}}$$

(2) $$G_{rat}=\frac{\sigma_{subj}^{2}+\sigma_{rep}^{2}+\sigma_{subj\_rep}^{2}}{\sigma_{subj}^{2}+\sigma_{rat}^{2}+\sigma_{rep}^{2}+\sigma_{subj\_rat}^{2}+\sigma_{subj\_rep}^{2}+\sigma_{rat\_rep}^{2}+\sigma_{residual}^{2}}$$

(3) $$G_{\mathrm{overall}\;\mathrm{test}}=\frac{\sigma_{\mathrm{subj}}^{2}}{\sigma_{\mathrm{subj}}^{2}+\frac{\sigma_{\mathrm{rat}}^{2}}{n_{\mathrm{rat}}}+\frac{\sigma_{\mathrm{rep}}^{2}}{n_{\mathrm{rep}}}+\frac{\sigma_{\mathrm{subj}\_\mathrm{rat}}^{2}}{n_{\mathrm{rat}}}+\frac{\sigma_{\mathrm{subj}\_\mathrm{rep}}^{2}}{n_{\mathrm{rep}}}+\frac{\sigma_{\mathrm{rat}\_\mathrm{rep}}^{2}}{n_{\mathrm{rep}}{*n}_{\mathrm{rat}}}+\frac{\sigma_{\mathrm{residual}}^{2}}{n_{\mathrm{rep}}{*n}_{\mathrm{rat}}}}$$

The significance level was set at alpha = 0.05.

## 3. Results

### 3.1. System Validity

**Figure 3:**
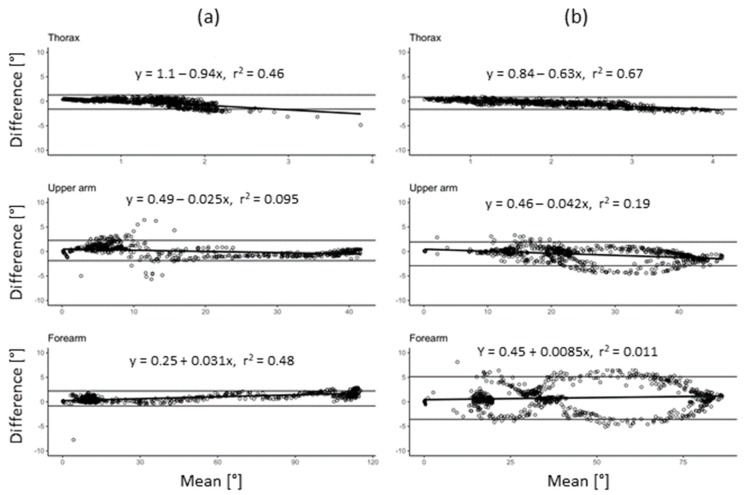
Bland–Altman analysis of the inertial and optical sensor systems. The data from one subject in one session of 10 repetitions. (**a**) finger-to-nose and (**b**) drinking task.

The system´s validity was assessed on one subject during the finger-to-nose and drinking tasks. The systematic error ranged between 0.25° and 1.1° for the former and 0.45 to 0.84 for the latter test (Figure 3a,b).

### 3.2. Inter-Rater and Within-Subject Reliability

**Figure 4:**
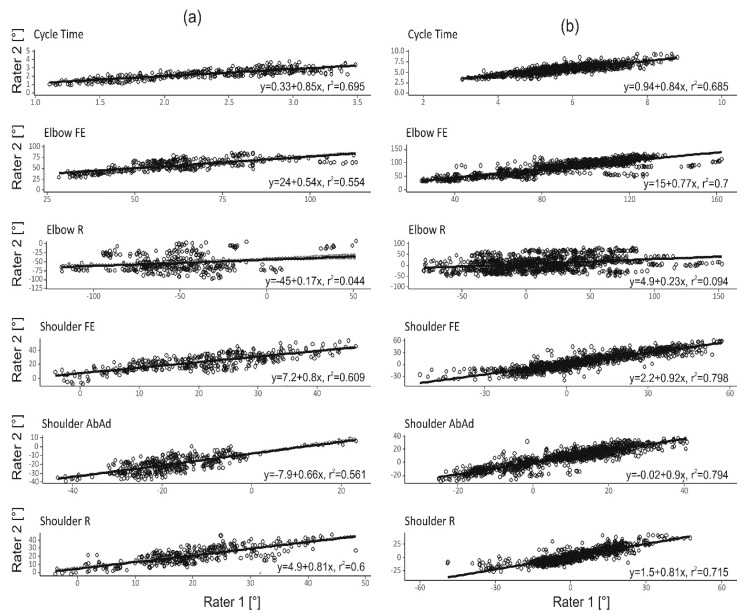
Illustration of inter-rater reliability between the two raters for the outcome measures of the (**a**) finger-to-nose and (**b**) drinking task. The regression lines are estimated using QR decomposition. Data of all 10 repetitions and 20 subjects are plotted.

**Figure 5:**
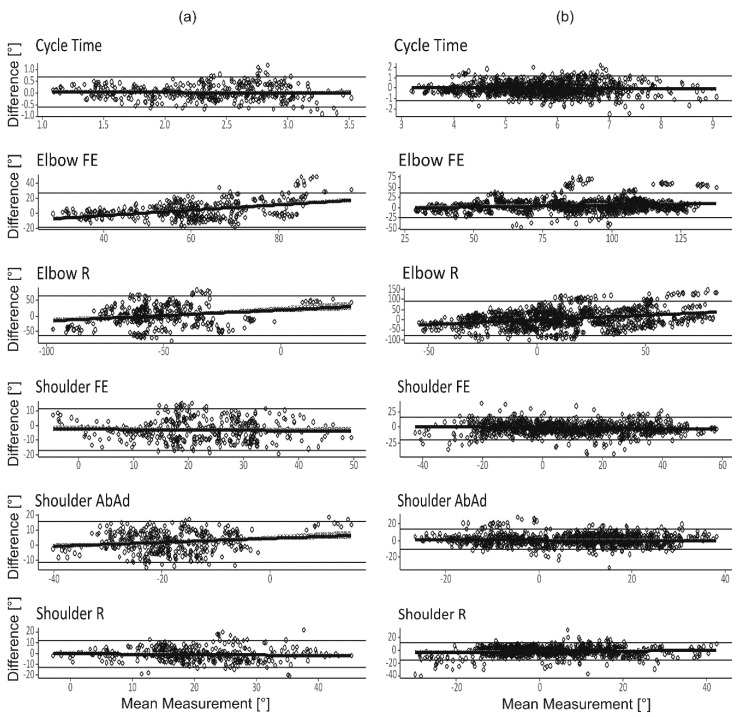
Bland–Altman analysis of the two raters for all outcome measures in the (**a**) finger-to-nose and (**b**) drinking task. Data of all 10 repetitions and 20 subjects are plotted.

Inter-rater reliability of the outcome measures cycle time and ROM are displayed in Figure 4 and Figure 5. R^2^ was above 50% for all outcome measures except for elbow R, where the R^2^ was low (<10%, Figure 4). In addition, for elbow R the linear regression coefficients were near zero, indicating a large variation in elbow axial rotation between the test sessions. Bland–Altman analyses (Figure 5), show that average discrepancy (i.e., bias) between raters was close to zero for all outcome measures, there was a dependency on mean angle for some of the ROM measures. The 95% confidence intervals of the Bland–Altman analyses were approximately 1.2 s for the cycle times, 15° for shoulder ROMs and 25° to 90° for elbow ROMs.

**Figure A1:**
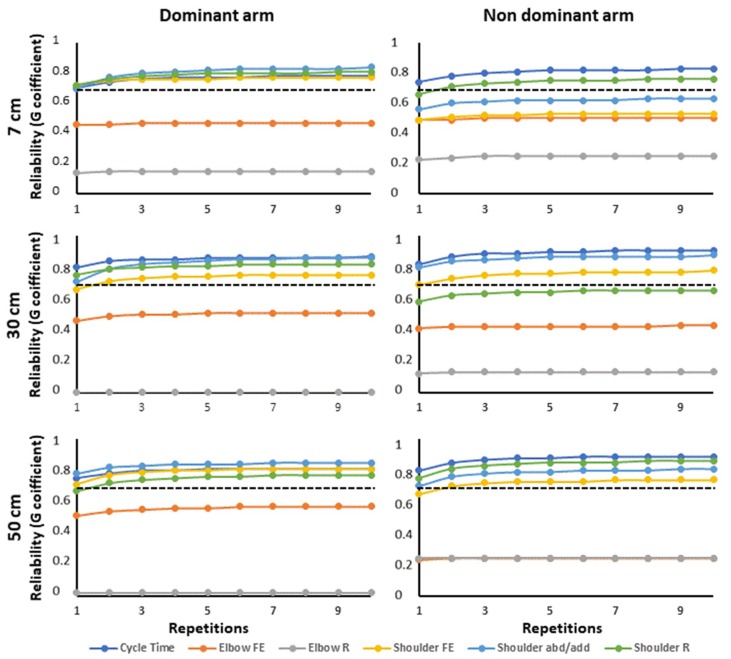
Illustrates the overall reliability (i.e., G-coefficient) at different distance to the glass, with different number of repetitions (assuming there is only one rater present) for the drinking task, with data from one rater. The results are subdivided into dominant arm (**left**) and non-dominant arm (**right**) for the different outcome measures. The dotted line marks the level for acceptable reliability; G = 0.7.

Based on the two LMM models, variance components and G-coefficients were calculated. Within-subject reliability was good to excellent for all measures of both tests and arms (Table 1 and Table 2). Results were the same for the distances 7 and 50 cm for the drinking task, although there was a small decrease in variability as distance increased (see Table A1 and Table A2 and Figure A1 in Appendix A).

Inter-rater reliability was acceptable to good for all measures except elbow R, shoulder AbAd (dominant arm) and elbow FE (non-dominant arm) for the finger-to-nose task. For the drinking task, inter-rater agreement was acceptable to good for all measures except the elbow ROMs, shoulder FE (dominant arm) and shoulder R (non-dominant arm).

The overall reliability of the tests, including both raters and 10 repetitions, was excellent for cycle time and good to excellent for all shoulder ROMs of both tests, except shoulder R for the non-dominant arm of the drinking task where it was acceptable. For the finger-to-nose task, elbow FE had an acceptable to good, and elbow R poor overall reliability. For the drinking task, reliability of all kinematic elbow measures was poor.

**Figure 6:**
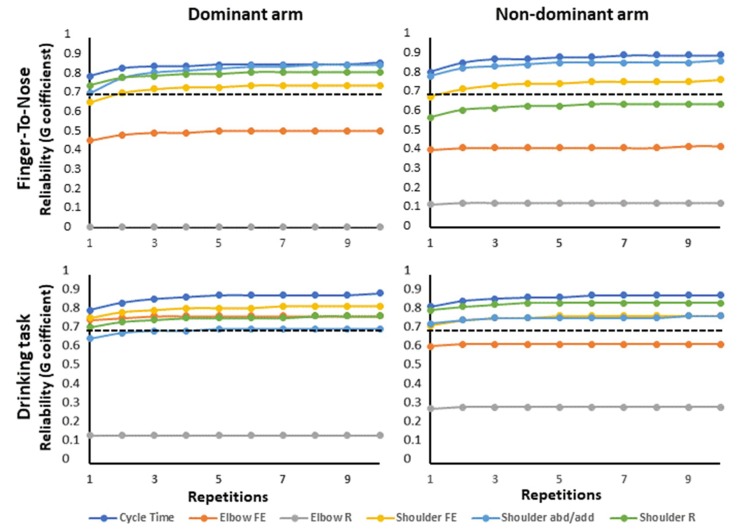
Illustrates the overall reliability (i.e., G-coefficient) estimated with a different number of repetitions for the finger-to-nose and drinking tasks based on one single rater. The results are subdivided into dominant arm (**left**) and non-dominant arm (**right**) for the different outcome measures. The dotted line marks the level for acceptable reliability, G = 0.7.

Figure 6 shows the G-coefficient as a function of the number of repetitions for one single rater. For both tests, three repeated measurements were enough to reach 95% of the final value obtained after 10 repetitions (Figure 6), but generally, the elbow ROMs did not reach an acceptable level of reliability regardless of the number of repetitions. 

## 4. Discussion

This study showed that objective kinematic outcome measures can be reliably collected with a portable sensor system. Such measures may offer added information to the assessment of movement function in the upper extremities. The system´s validity against a gold standard optical reference system was verified, however, the reliability depended on movement direction and type of joint: elbow ROMs had a higher inter-rater variability, while cycle time and shoulder ROMs were reliably assessed.

Both ROM and cycle time are clinically relevant outcome measures, e.g., ROM is often affected in neurological disorders [13,28], while a prolonged cycle time is used to indicate impaired coordination [15,29]. Within generalizability theory [26], G-coefficient is analogous to the reliability coefficient, intra-class correlation (ICC), in classical test theory. In contrast to ICC, G-coefficient accounts for different sources of systematic and random errors and estimates them individually [20]. By incorporating different variance components into the error variance when calculating G, specific effects of factors, e.g., measurement repetitions within subjects and differences between raters, can be estimated. Based on G theory it is also possible to determine the optimal study design [20]. In our study, this was applied to demonstrate the number of repetitions needed to achieve the specified overall reliability.

### 4.1. Within-Subject Reliability

Within-subject reliability was good to excellent (0.89–0.97 and 0.80–0.96) for all outcome measures of the finger-to-nose and drinking task, respectively. Thus, healthy subjects perform repetitive arm movements (during one single session) with a consistent movement pattern. Drinking task is widely used in clinical practice, but the performance varies between applications. To evaluate whether the distance chosen was important for the overall reliability, thee distances (close to far) were investigated. Previous studies show a variety in the distance utilized [10,11,30] or distances inadequately described [31]. Results indicate a small decrease in variability as distance increases (Figure A1, Appendix A), which enhances the importance of choosing a standardized distance to be able to compare between studies and occasions.

### 4.2. Rater Dependency

In general, inter-rater reliability for the finger-to-nose task was acceptable for all outcome measures except elbow R. For the drinking task it was poor for all elbow ROMs, and good or close to acceptable for all other measures. Both Bland–Altman plots and LMM analyses show a small systematic difference between raters (Figure 5 and σ^2^_rat_ in Table 2), indicating that standardization of sensor placement was adequate. The largest source of error was the random variance due to the interaction between rater and subject (σ^2^_sub_rat_ Table 2), especially for elbow R. This was also shown by the low R^2^ in the linear regression analysis (Figure 4) and large confidence intervals in the Bland–Altman plots (Figure 5). We speculate that high rater/subject interaction for elbow ROMs could have different explanations. (1) Difficulties in completely standardizing the test. Despite recorded instructions which informed participants to start with the hand in a pronated position, it was observed that starting position varied between forearm supination and pronation for some participants. (2) The elbow must be flexed to a certain degree to reach the nose or glass, but the amount of rotation may vary while still being able to fulfill the test. This well-known variability was described previously [32].

For all outcome measures, test-retest variations of the participants must be considered a source affecting the subject/rater variation. Since each rater only assessed each subject on one occasion, test-retest variation due to natural subject variations could not be estimated, i.e., the same rater tests the same participant at a later occasion. 

Cycle time had higher within-subject, inter-rater, and overall reliability than all kinematic outcome measures. This finding was not surprising since goal-directed movements can be performed along different trajectories. Hence different combinations of joint angles can be used to reach the same target [32], which results in a larger variability in joint rotation compared to temporal measures.

### 4.3. Proposed Protocol Design for Increased Reliability

The simplicity of performing multiple measurements when using portable motion sensors encourages the strategy of applying repeated measurements to improve estimation reliability. Figure 6 shows the overall improvement in measurement reliability as a function of repetitions. It is evident that only a few repetitions are needed, due to low within-subject variability. 

Even though the overall reliability was high the accuracy could be improved if the number of raters assessing each participant had been increased, i.e., the ratio σ^2^_subj_rat_/n_rat_ will decrease as n_rat_ increases since σ^2^_subj_rat_ is large (Equation (3), Table 1 and Table 2). This is also evident from higher overall reliability in Table 1 and Table 2 (two raters) compared to Figure 2 (one rater). Unfortunately, in a clinical situation, this is seldom possible due to practical limitations. However, often it is the relative change over time or comparison with a normal range, rather than the absolute value, that is meaningful for clinical diagnoses/treatment.

### 4.4. Kinematic Assessment of the Finger-To-Nose Task

Recent studies, also applying different versions of the finger-to-nose task, found total movement time to be the strongest indicator of stroke impairment (mean difference between the control and stroke groups was 2.6 s) [13] and to discriminate between mild and moderate-to-severe impairment post-stroke (cut-off level in total movement time was 10.6 s) [33]. This shows the clinical usefulness of this parameter, and since cycle time had excellent overall reliability and low between-subject variance (σ^2^_subj_ = 0.29 s (non-dominant arm), Table 1) the current setup is well suited for detecting impairment post-stroke. The high reliability is consistent with a previous study, where the mean cycle time during a finger-to-nose task was 2.44 s with a corresponding ICC = 0.82 [34].

In order to quantify subtle changes during e.g., rehabilitation it is important, however, to extend the information extracted from the finger-to-nose task beyond cycle time [13]. Shoulder ROMs, reliably assessed in the present study, have been shown to be valuable in the treatment and diagnosis of neurological disorders, e.g., in a group of patients with stroke impairments, shoulder FE/AbAd were used to show improvements after training with a specific rehabilitation device (change in FE: ~35° and Ab-Ad: ~23°) [35]. Shoulder ROMs were also used to investigate upper limb mobility in patients with different types of spinal cord injuries [36]. 

Further, elbow FE significantly separated between persons with mild and moderate post-stroke symptoms (group mean; mild symptoms 74.3° vs. moderate symptoms 59.9°) when measured with an optical measurement system [13]. This strengthens the clinical value of adding elbow FE to the clinical protocols. Unfortunately, elbow FE only had poor to acceptable overall reliability, stressing the need for further standardization of the measurement protocols when using inertial measurement unit (IMU) systems.

### 4.5. Kinematic Assessment of the Drinking Task

Like the finger-to-nose task, cycle time was the most repeatable parameter with highest inter-rater reliability. Alt Murphy et al. demonstrated that a decreased movement time of 2.5 to 5 s marks a clinical improvement post-stroke [29], which is easily detectable with our measurement setup (95% confidence interval for Bland–Altman analysis ± 1.2 s). Also, movement time discriminated between persons with a spinal injury on C6 level and healthy controls (group mean difference 2.6 s) [11], stressing the clinical value of measuring cycle time.

Kinematic measures assessed by opto-electronic motion caption systems have also been found to give additional and more sensitive information about upper extremity movement function during the drinking task [9,10,11], emphasizing the need to be able to reliably quantify these parameters also in a clinical setting. A pilot study on post stroke patients indicated decreased ROM in shoulder FE (group mean difference 20.5°) and increased ROM in shoulder AbAd (group mean difference 15.2°) [28]. Such minor changes in ROM are hard to see by visual inspection but may be reliably detected with the present sensor system (overall reliability including one rater and 10 repetitions, G = 0.75–0.88, Figure 6).

### 4.6. Methodological Considerations and Strengths

High validity of the sensor system was confirmed in the current test situations. A deviation of 0.25° and 1.1° from the optical reference system is within the range expected and small compared to the kinematic measures assessed.

Since repeatability and measurement errors represent two important but different aspects of overall reliability, G-coefficients, as well as Bland–Altman analyses, were applied [26,37]. The test re-test variability was evaluated using two different raters, as opposed to the same rater evaluating all participants at a later occasion. Since the variability among raters was high, we believe that the precision would improve even further if one single rater had performed the test. We, therefore, recommend that, if possible, the same clinician re-evaluate the patient during a rehabilitation process. If this is not possible, the variability due to different raters needs to be considered. Also, it is important that the test protocol is standardized. The high reliability in most outcomes was probably related to the strictly followed protocol when mounting the sensors, and standardization of verbally recorded instructions.

To get a more complete picture of the complex function of the upper extremities, future studies should focus on additional measures e.g., velocity and movement smoothness. It is also important to include patients with various diseases and healthy elderly people, rather than only healthy young individuals, as individual variability may differ with condition which in turn affects the applicability of a chosen measurement technique. Variability in kinematical measures can also be used on its own to detect deficiencies in motor function [38].

## 5. Conclusions

In conclusion, the reliability of the kinematic measures depended on movement direction and joint. The range in elbow joint axial rotation had poor reliability while the range in elbow flexion-extension was close to acceptable. Standardized measurement procedures are important to minimize confounding factors. Still, the high variability could also be due to natural movement variations in this joint. The within-subject variability may serve as a baseline when assessing the upper extremities in future patient groups. Cycle time and shoulder range of motion showed good to excellent reliability and are suitable to incorporate in the drinking and finger-to-nose tasks. The inertial sensors provided good to excellent measures of cycle time and shoulder ROM in non-disabled individuals and thus have the potential to improve today’s assessment of arm function. 

## Figures and Tables

**Table 1:** Linear mixed effect model for the finger-to-nose task.

	Side	Reliability	Cycle Time	Elbow FE	Elbow R	Shoulder FE	Shoulder AbAd	Shoulder R
	Dominant	Within-subject	0.89	0.91	0.97	0.89	0.92	0.91
		Inter-rater	0.84	0.77	0.15	0.78	0.67	0.73
G-coefficients		Overall	0.94	0.88	0.26	0.91	0.83	0.87
	Non-dominant	Within-subject	0.91	0.94	0.96	0.90	0.92	0.94
		Inter-rater	0.83	0.62	0.30	0.74	0.76	0.82
		Overall	0.94	0.77	0.47	0.88	0.87	0.92
	Dominant	Intercept	2.35 s ***	58.66° ***	−53.66° ***	22.42° ***	−20.08° ***	20.72° ***
	*n* = 397		(0.12)	(4.14)	(4.70)	(2.81)	(1.72)	(1.89)
		σ^2^_rat_	0.00	13.71	0.00	3.99	0.00	0.29
		σ^2^_rep_	0.00	0.00	5.90	0.76	0.62	0.14
		σ^2^_subj_	0.28	186.75	113.81	107.42	48.16	60.17
Model and		σ^2^_rep_rat_	0.00	0.00	0.00	0.00	0.00	0.00
variance		σ^2^_subj_rat_	0.03	37.48	629.23	17.02	18.76	16.16
components		σ^2^_subj_rep_	0.01	2.72	1.10	2.20	0.57	0.92
		σ^2^_residual_	0.02	6.32	14.05	8.43	4.55	6.49
	Non-dominant	Intercept	2.31 s ***	61.35° ***	−54.47° ***	21.87° ***	−16.26° ***	19.94° ***
	*n* = 379		(0.13)	(4.04)	(4.14)	(2.64)	(2.69)	(2.13)
		σ^2^_rat_	0.00	9.61	0.00	3.57	4.35	0.00
		σ^2^_rep_	0.00	0.30	3.94	0.02	0.00	0.06
		σ^2^_subj_	0.29	173.26	148.50	87.90	85.66	79.07
		σ^2^_rep_rat_	0.00	0.00	0.00	0.14	0.09	0.00
		σ^2^_subj_rat_	0.03	90.53	338.92	20.29	19.94	12.80
		σ^2^_subj_rep_	0.00	1.89	2.41	1.24	1.60	1.42
		σ^2^_residual_	0.03	4.74	14.53	6.52	3.47	4.69

*** *p* < 0.001, ** *p* < 0.01, * *p* < 0.05. Statistical model: Response ~ (1 | “Subject”) + (1 | “Rater”) + (1 | “Repetition”) + (1 | “Subject”:”Repetition”) + (1 | “Subject”:”Rater”) + (1 | “Repetition”:”Rater”). The dataset was stratified using the factor “arm dominance”. In the table, σ^2^_sub_, σ^2^_rat,_ σ^2^_rep__,_ σ^2^_rep_rat,_ σ^2^_subj_rat,_ σ^2^_subj_rep,_ and σ^2^_residual_ are the variance between subjects, among raters, among repetitions, of the interaction between repetitions and raters, subjects and raters, subjects and repetitions, and residuals respectively. Values within parentheses indicate the mean square error of each parameter estimate. The reliability estimates (defined by the G-coefficient, absolute agreement) for the different responses are subdivided into within-subject, inter-rater, and overall reliability. The number of subjects = 20 and the number of raters = 2.

**Table 2:** Linear mixed effect model for the drinking task at 30 cm distance.

	Side	Reliability	Cycle Time	Elbow FE	Elbow R	Shoulder FE	Shoulder AbAd	Shoulder R
	Dominant	Within-subject	0.92	0.88	0.97	0.85	0.80	0.90
		Inter-rater	0.81	0.47	0.002	0.67	0.72	0.78
G-coefficients		Overall	0.93	0.68	0	0.86	0.93	0.90
	Non-Dominant	Within-subject	0.90	0.96	0.96	0.87	0.90	0.87
		Inter-rater	0.84	0.42	0.14	0.72	0.81	0.60
		Overall	0.95	0.60	0.23	0.87	0.93	0.79
	Dominant	Intercept	5.65 s ***	90.76° ***	20.09° ***	5.68° **	14.69° ***	−5.00° **
	*n* = 397		(0.20)	(2.77)	(5.62)	(1.76)	(1.64)	(1.68)
		σ^2^_rat_	0.00	0.00	0.00	0.00	0.91	0.00
		σ^2^_rep_	0.00	2.38	0.00	0.16	0.00	1.04
		σ^2^_subj_	0.71	100.31	0.00	53.09	41.88	48.74
Model and		σ^2^_rep_rat_	0.00	0.88	0.00	0.00	0.14	0.00
variance		σ^2^_subj_rat_	0.10	92.30	1260.94	16.06	4.53	10.01
components		σ^2^_subj_rep_	0.01	0.00	2.08	1.22	0.57	0.77
		σ^2^_residual_	0.06	23.05	36.25	10.46	11.02	4.44
	Non-dominant	Intercept	5.68 s ***	97.79° ***	24.75° ***	2.94°	15.84° ***	−3.15°
	*n* = 376		(0.19)	(2.96)	(5.38)	(2.37)	(1.74)	(1.62)
		σ^2^_rat_	0.00	0.00	0.00	0.22	0.00	0.00
		σ^2^_rep_	0.00	0.03	3.67	0.00	0.00	0.00
		σ^2^_subj_	0.62	99.10	124.19	90.38	53.51	39.08
		σ^2^_rep_rat_	0.00	0.40	0.00	0.82	0.09	0.21
		σ^2^_subj_rat_	0.05	133.03	832.17	24.28	6.91	19.92
		σ^2^_subj_rep_	0.01	1.36	9.89	4.21	0.68	1.33
		σ^2^_residual_	0.07	7.43	23.90	11.52	5.94	7.22

*** *p* < 0.001, ** *p* < 0.01, * *p* < 0.05. Statistical model: Response ~ (1 | “Subject”) + (1 | “Rater”) + (1 | “Repetition”) + (1 | “Subject”:”Repetition”) + (1 | “Subject”:”Rater”) + (1 | “Repetition”:”Rater”). The dataset was stratified using the factor “arm dominance”. In the table, σ^2^_sub_, σ^2^_rat,_ σ^2^_rep,_ σ^2^_rep_rat,_ σ^2^_subj_rat,_ σ^2^_subj_rep,_ and σ^2^_residual_ are the variance between subjects, among raters, among repetitions, of the interaction between repetitions and raters, subjects and raters, subjects and repetitions, and residuals respectively. Values within parentheses indicate the mean square error of each parameter estimate. The reliability estimates (defined by the G-coefficient, absolute agreement) for the different responses are subdivided into within-subject, inter-rater, and overall reliability. The number of subjects = 20 and the number of raters = 2.

## Appendix A

**Table A1:** Linear mixed effect model for the drinking task at 7 cm distance.

	Side	Reliability	Cycle Time	Elbow FE	Elbow R	Shoulder FE	Shoulder AbAd	Shoulder R
	Dominant	Within-subject	0.88	0.97	0.93	0.93	0.82	0.88
		Inter-rater	0.72	0.46	0.15	0.73	0.81	0.74
G-coefficients		Overall	0.88	0.64	0.26	0.87	0.90	0.89
	Non-dominant	Within-subject	0.88	0.97	0.93	0.90	0.88	0.86
		Inter-rater	0.75	0.49	0.25	0.50	0.59	0.67
		Overall	0.90	0.67	0.40	0.69	0.77	0.86
	Dominant	Intercept	5.11 ***	56.22 ***	−12.61 *	29.97 ***	−8.33 ***	12.15 ***
			(0.18)	(4.80)	(4.96)	(2.72)	(1.98)	(2.45)
	*n* = 399	σ^2^_rat_	0.00	20.72	0.00	3.32	0.00	2.33
		σ^2^_rep_	0.01	0.97	0.48	0.00	7.53	0.36
		σ^2^_subj_	0.58	167.39	128.74	101.04	57.90	86.98
Model and		σ^2^_rep_rat_	0.00	0.00	0.00	0.41	0.00	0.00
variance		σ^2^_subj_rat_	0.15	166.03	717.93	25.69	9.82	17.05
components		σ^2^_subj_rep_	0.01	1.55	4.79	1.19	1.22	2.13
		σ^2^_residual_	0.08	10.10	60.34	8.84	5.92	12.41
	Non-dominant	Intercept	5.11 ***	60.41 ***	−13.04 **	29.66 ***	−4.29 *	15.13 ***
	*n* = 377		(0.17)	(4.53)	(4.40)	(2.53)	(2.17)	(1.99)
		σ^2^_rat_	0.00	17.59	0.00	2.75	1.59	0.00
		σ^2^_rep_	0.01	0.31	1.51	0.00	0.51	0.00
		σ^2^_subj_	0.51	154.37	146.22	67.17	56.97	64.87
		σ^2^_rep_rat_	0.00	0.00	0.32	0.26	0.10	0.00
		σ^2^_subj_rat_	0.10	134.95	433.00	54.84	30.80	19.13
		σ^2^_subj_rep_	0.00	1.45	8.57	1.65	2.91	0.68
		σ^2^_residual_	0.07	8.45	32.93	11.19	9.16	13.18

*** *p* < 0.001, ** *p* < 0.01, * *p* < 0.05. Statistical model: Response ~ (1 | “Subject”) + (1 | “Rater”) + (1 | “Repetition”) + (1 | “Subject”:”Repetition”) + (1 | “Subject”:”Rater”) + (1 | “Repetition”:”Rater”). The dataset was stratified using the factor “arm dominance”. In the table, σ^2^_sub_, σ^2^_rat,_ σ^2^_rep,_ σ^2^_rep_rat,_ σ^2^_subj_rat,_ σ^2^_subj_rep,_ and σ^2^_residual_ are the variance between subjects, among raters, among repetitions, of the interaction between repetitions and raters, subjects and raters, subjects and repetitions, and residuals respectively. Values within parentheses indicate the mean square error of each parameter estimate. The reliability estimates (defined by the G-coefficient, absolute agreement) for the different responses are subdivided into within-subject, inter-rater, and overall reliability. The number of subjects = 20 and the number of raters = 2.

**Table A2:** Linear mixed effect model for the drinking task at 50 cm distance.

	Side	Reliability	Cycle Time	Elbow FE	Elbow R	Shoulder FE	Shoulder AbAd	Shoulder R
	Dominant	Within-subject	0.91	0.87	0.98	0.86	0.91	0.86
		Inter-rater	0.78	0.51	0.02	0.72	0.79	0.70
		Overall	0.90	0.73	0.04	0.90	0.92	0.87
G-coefficients								
	Non-dominant	Within-subject	0.89	0.94	0.97	0.87	0.85	0.86
		Inter-rater	0.83	0.25	0.24	0.67	0.73	0.76
		Overall	0.94	0.39	0.39	0.85	0.89	0.92
	Dominant	Intercept	6.32 ***	106.47 ***	17.23 **	−8.03 ***	15.69 ***	−3.65 *
	*n* = 395		(0.21)	(2.58)	(5.91)	(2.36)	(1.83)	(1.75)
		σ^2^_rat_	0.00	3.17	0.00	0.00	0.00	0.58
		σ^2^_rep_	0.00	0.02	0.00	0.13	0.00	1.25
		σ^2^_subj_	0.77	74.92	25.21	100.03	61.76	46.20
		σ^2^_rep_rat_	0.00	0.00	1.48	0.00	0.00	0.00
Model and		σ^2^_subj_rat_	0.17	51.27	1342.60	20.93	10.28	12.11
variance		σ^2^_subj_rep_	0.03	0.00	4.31	0.00	0.49	0.38
components		σ^2^_residual_	0.06	18.56	24.63	18.96	6.63	8.23
	Non-dominant	Intercept	6.28 ***	114.38 ***	29.04 ***	−11.99 ***	16.16 ***	−4.74 **
	*n* = 377			(0.19)	(3.86)	(6.40)	(2.56)	(1.77)
		σ^2^_rat_	0.00	20.71	0.00	1.20	0.00	0.08
		σ^2^_rep_	0.00	0.23	3.11	0.00	0.00	0.30
		σ^2^_subj_	0.62	37.73	302.47	97.08	53.01	48.27
		σ^2^_rep_rat_	0.00	0.00	0.80	0.00	0.12	0.00
		σ^2^_subj_rat_	0.07	94.76	935.13	30.81	11.60	6.90
		σ^2^_subj_rep_	0.02	3.66	5.39	2.83	2.25	0.18
		σ^2^_residual_	0.07	6.41	22.83	16.34	8.68	8.61

*** *p* < 0.001, ** *p* < 0.01, * *p* < 0.05. Statistical model: Response ~ (1 | “Subject”) + (1 | “Rater”) + (1 | “Repetition”) + (1 | “Subject”:”Repetition”) + (1 | “Subject”:”Rater”) + (1 | “Repetition”:”Rater”). The dataset was stratified using the factor “arm dominance”. In the table, σ^2^_sub_, σ^2^_rat,_ σ^2^_rep,_ σ^2^_rep_rat,_ σ^2^_subj_rat,_ σ^2^_subj_rep,_ and σ^2^_residual_ are the variance between subjects, among raters, among repetitions, of the interaction between repetitions and raters, subjects and raters, subjects and repetitions, and residuals respectively. Values within parentheses indicate the mean square error of each parameter estimate. The reliability estimates (defined by the G-coefficient, absolute agreement) for the different responses are subdivided into within-subject, inter-rater, and overall reliability. The number of subjects = 20 and the number of raters = 2.
